# Safety and pharmacokinetics of imaradenant (AZD4635) in Japanese patients with advanced solid malignancies: a phase I, open-label study

**DOI:** 10.1007/s00280-023-04605-9

**Published:** 2023-12-13

**Authors:** Nobuaki Matsubara, Shota Kusuhara, Noboru Yamamoto, Kazuki Sudo, Masahiko Yanagita, Kosho Murayama, Hisashi Kawasumi, Deanna L. Russell, Da Yin, Toshio Shimizu

**Affiliations:** 1 Department of Medical Oncology, National Cancer Center Hospital East, 6-5-1 Kashiwanoha, Kashiwa, Chiba 277-8577 Japan; 2 Department of Experimental Therapeutics, National Cancer Center Hospital, Tokyo, Japan; 3 Research & Development, AstraZeneca K.K., Tokyo, Japan; 4 Research & Development, AstraZeneca K.K., Osaka, Japan; 5 Translational Medicine, Early Oncology, Oncology R&D, AstraZeneca Pharmaceuticals, Boston, MA USA; 6 Oncology Data Science, Research and Early Development, Oncology R&D, AstraZeneca Pharmaceuticals, Gaithersburg, MD USA; 7 Present Address: Oncology Medical Affairs, Pfizer Japan Inc., Tokyo, Japan; 8 Present Address: Department of Pulmonary Medicine and Medical Oncology, Wakayama Medical University Graduate School of Medicine, Wakayama Medical University Hospital, Wakayama, Japan

**Keywords:** Adenosine A2A receptor antagonists, Immunotherapy, Neoplasms, Pharmacokinetics, Safety

## Abstract

**Purpose:**

Imaradenant is a novel potent and selective adenosine A2A receptor antagonist that is hypothesized to reduce immune suppression in the tumor microenvironment. This phase I, open-label, dose-escalation study evaluated the safety, pharmacokinetics, and anti-tumor activity of imaradenant.

**Methods:**

Japanese patients with advanced solid malignancies received imaradenant 50 mg (*n* = 3) or 75 mg (*n* = 7) once daily (QD). The primary objective was safety and tolerability, and the secondary objectives were pharmacokinetics and anti-tumor activity.

**Results:**

The median treatment duration was 2.10 months and 2.14 months for the 50- and 75-mg QD cohorts, respectively. The most common adverse events were nausea, malaise, decreased appetite, and vomiting. Five patients (50%) reported adverse events that were considered causally related to imaradenant; three patients had Grade 2 adverse events of malaise, nausea, and diarrhea. No deaths or serious adverse events occurred. The median times of maximum observed concentrations sampled after a single dose in the 50- and 75-mg QD cohorts were 1.08 h (range, 0.95–1.95) and 2.00 h (range, 0.92–5.52), respectively. There was little accumulation after multiple dosing, with geometric mean accumulation ratios of maximum concentration of 1.3 (50-mg QD) to 1.4 (75-mg QD) and area under the concentration–time curve 0–24 of 1.4 (50-mg QD) to 1.5 (75-mg QD). The best objective response was stable disease (3/10).

**Conclusion:**

No new or unexpected safety concerns were identified, and imaradenant had an acceptable safety profile at both 50- and 75-mg QD.

**ClinicalTrials.gov identifier** NCT03980821 (June 10, 2019).

**Supplementary Information:**

The online version contains supplementary material available at 10.1007/s00280-023-04605-9.

## Introduction

Despite advances in cancer immunotherapy, the efficacies of different types of immunotherapies such as adoptive cell transfer and immune checkpoint inhibitors vary, and not all patients will benefit from these treatments [1, 2]. Adenosine is one of several metabolic breakdown products in the tumor microenvironment (TME) that can suppress an immune response to limit tissue injury [3], and next-generation immuno-oncology therapeutics include the development of inhibitors of extracellular adenosine (eADO) signaling.

In one of the pathways leading to eADO production, extracellular AMP (eAMP) is produced and hydrolyzed to eADO by cluster of differentiation (CD) 73 (encoded by the gene 5′-nucleotidase ecto [*NT5E*]) [2]. Additionally, other membrane-bound phosphatases such as tissue-specific and tissue-non-specific alkaline phosphatases hydrolyze eAMP to eADO. The activation of adenosine A2A and A2B receptors (A_2A_R/A_2B_R) suppresses the anti-tumor activity of tumor-infiltrating immune cells. Furthermore, activation of A_2B_ signaling in tumor cells supports the survival and metastasis of these tumor cells [2]. In preclinical models, the targeted inhibition of CD73, CD39, CD38, A_2A_, or A_2B_ has re-established anti-tumor immunity and improved the efficacy of cancer immunotherapies [2].

Imaradenant (formerly AZD4635) is a novel and potent selective A_2A_R antagonist that has been developed for the treatment of cancer, which blocks the ability of adenosine to bind A_2A_R in a dose-dependent manner. It is hypothesized that A_2A_R receptor blockade in humans will lead to decreased immune suppression in the TME [4, 5]. Such modulation of the TME may allow a more robust anti-tumor immune response. A phase Ia/b trial in the USA showed that therapy with imaradenant in combination with durvalumab, an immune checkpoint inhibitor, was well tolerated in patients with advanced solid tumors [5]. However, the safety of imaradenant monotherapy has not been determined in Japanese patients. To determine whether ethnic differences may impact the safety or tolerability of a new drug, the Japanese regulatory authority requires phase I trials of new drugs to be conducted so that, if necessary, modifications may be applied to future study designs specific to Japanese patients in the clinical development process [6]. Therefore, we conducted this first-in-Japan phase I trial.

Our primary objective was to assess the safety and tolerability of imaradenant in Japanese patients with advanced solid malignancies. The secondary objectives were to evaluate the pharmacokinetic (PK) profile and anti-tumor activity of imaradenant in this patient population, and an additional exploratory objective was to evaluate the biomarker status of patients treated with imaradenant. The results from this study will form the basis for decisions for future studies.

## Materials and methods

### Study design and treatment

This phase I, open-label, dose-escalation study was conducted at the National Cancer Center Hospital (Tokyo, Japan) and the National Cancer Center Hospital East (Chiba, Japan) between July 2019 and September 2020. The study consisted of two cohorts: the imaradenant 50-mg once daily (QD) cohort and the imaradenant 75-mg QD cohort (Table S1 in Online Resource 1). At least three and up to six evaluable Japanese patients with advanced solid malignancies were planned to be enrolled in the 50-mg QD cohort and six evaluable patients were required for the 75-mg QD cohort to confirm the tolerability of imaradenant. The total number of evaluable patients in each cohort depended upon the available data in each cohort and the decision of the Safety Review Committee; each cohort could be expanded to include a maximum of 12 patients to further assess the PK or safety of imaradenant.

Patients received the designated dose of imaradenant QD, with Cycle 0 defined as the time from the first single administration of imaradenant given on Day 1 followed by 3 days off (from Day 2 to Day 4) to evaluate the PK characteristics after a single-dose administration. Subsequent cycles were defined as 21 days of continuous administration, and patients continued treatment until the discontinuation criteria were met. The protocol was approved by the National Cancer Center Institutional Review Board, and the study was conducted in accordance with the Declaration of Helsinki and adhered to Good Clinical Practice guidelines. All patients provided written informed consent. This study was registered at ClinicalTrials.gov under the identifier NCT03980821.

### Patients

The inclusion criteria were as follows: Japanese patients ≥ 20 years of age at study entry; histological or cytological confirmation of a solid, malignant tumor that was refractory to standard therapies or for which no standard therapies existed; at least one lesion evaluable at baseline or a measurable prostate-specific antigen level above normal limits; an ECOG performance status of 0 to 1; and normotensive or well controlled blood pressure (< 140/90 mmHg).

The exclusion criteria were as follows: patients who had received nitrosourea or mitomycin C within 6 weeks of the first dose of study treatment; any investigational medicinal product or other systemic anticancer treatment within 4 weeks of the first dose; cytochrome P450 enzyme 1A2 (CYP1A2) typical substrates, potent or moderate inducers/inhibitors of CYP1A2, or typical substrates of breast cancer resistance protein and organic anion transporter 1 that could not be discontinued by 2 weeks prior to the first administration of imaradenant treatment; prior therapy with imaradenant or any other A_2A_R antagonist; or if there was evidence of any significant cardiovascular disease or any other relevant disease or disorder.

### Endpoints

The safety endpoints included adverse events (AEs), serious AEs (SAEs), dose-limiting toxicities (DLTs), vital signs, cardiac function (electrocardiogram [ECG] and echography/multigated acquisition scan [ECHO/MUGA] results), and laboratory parameters.

Plasma concentrations of imaradenant and its metabolites (SSP-005174 [active] and SSP-005173 [inactive]) after single and multiple administration of imaradenant 50 mg and 75 mg were assessed for PK analysis. For single administration of imaradenant, plasma concentrations were determined at pre-dose and 0.5, 1, 2, 4, 6, 8, 24, 48, and 72 h post-dose on Cycle 0, Day 1. For multiple administration of imaradenant, plasma concentrations were determined at pre-dose on Cycle 1, Day 1; at pre-dose and 0.5, 1, 2, 4, 6, 8, and 24 h post-dose on Cycle 1, Day 15; and at pre-dose on Day 1 of even-numbered cycles.

The following endpoints were evaluated to assess the anti-tumor activity of imaradenant: objective response rate (ORR), disease control rate (DCR), duration of response (DoR), and progression-free survival (PFS), assessed by RECIST v1.1. Exploratory objectives included the assessment of baseline biomarker status and molecular responses to imaradenant treatment for any association with clinical response. This included the evaluation of intra-tumoral and peripheral gene expression, immune composition, and tumor genetics.

### Statistical methods

Nine to 24 evaluable patients were planned to be enrolled in the study. Safety analyses were performed using the safety analysis set, defined as all patients who received at least one dose of imaradenant treatment. AEs were coded by system organ class (SOC) and preferred term (PT) using the Medical Dictionary for Regulatory Activities (MedDRA) version 21.1 or higher and graded by Common Terminology Criteria for Adverse Events (CTCAE) version 5.0. AEs occurring within the defined 30-day follow-up period after discontinuation of imaradenant treatment were included in the AE summaries. AEs occurring before the first administration of imaradenant treatment were included in the listings, but excluded from the summary tables of AEs. Any AEs that occurred after a patient received further therapy for cancer (following discontinuation of imaradenant treatment) during the 30-day follow-up period were flagged in the data listings. AEs occurring after the 30-day follow-up period after discontinuation of imaradenant treatment were listed separately, but not included in the summaries. Raw values and changes from baseline for hematology, clinical chemistry, ECG, ECHO/MUGA, and vital signs were summarized using descriptive statistics (mean, median, standard deviation (SD), minimum, maximum, and number of observations) by cohort and overall. The CTCAE grade was summarized for laboratory variables included in the revised CTCAE version 5.0. DLTs were evaluated using the DLT analysis set, defined as all patients who received at least 75% of imaradenant treatment during Cycles 0 and 1, or all patients who had a DLT during the DLT assessment period.

The PK analysis set was defined as all patients who received at least one administration of imaradenant treatment with at least one reportable concentration; however, if there were important AEs or protocol deviations that may have impacted the PK, an additional analysis that excluded patients with those occurrences was conducted. Plasma concentrations of imaradenant and metabolites were summarized by nominal sample time, cohort (e.g., dose level), and by visit and day. Derived PK parameters were summarized by cohort. Concentrations and derived PK parameters were reported using descriptive statistics.

The tumor response analysis set was defined as all dosed patients with a baseline tumor assessment or new lesion per RECIST v1.1. The best objective response (BOR; categorized as complete response [CR], partial response [PR], stable disease, progressive disease [PD], and not evaluable [NE]), ORR, and DCR were summarized based on RECIST v1.1 by cohort and overall using the tumor response analysis set. Target lesion size at each tumor assessment time point was summarized, along with percentage change from baseline. The best percentage change in tumor size from baseline over all tumor assessment time points was summarized using descriptive statistics. The DoR was listed for patients who had a confirmed response. PFS based on RECIST v1.1 was assessed and listed for patients in the safety analysis set. Biomarker analysis was evaluated using the biomarker analysis set, defined as all patients that participated in the exploratory biomarker research. All statistical analyses were performed using SAS software version 9.4 (SAS Institute Inc., Cary, NC, USA).

### Biomarker analysis

#### Circulating tumor DNA (ctDNA) analysis

Twenty-eight plasma samples from ten patients across three time points (baseline, Cycle 3/Day 1, and end of treatment) were sent to Guardant Health, where ctDNA isolation, targeted sequencing on the Guardant360 panel (Guardant, Redwood City, CA, USA), and variant calling were performed. Potential germline mutations and clonal hematopoiesis of indeterminate potential (CHIP) variations were filtered and the mean variant allele frequency (VAF) for somatic genomic alterations was calculated for each sample as previously described [7].

#### T cell receptor (TCR) sequencing

Baseline tumor formalin-fixed paraffin-embedded (FFPE) samples from six patients and baseline blood samples from five patients were sent to Adaptive Biotechnologies (Seattle, WA, USA) for genomic DNA extraction and immunosequencing of the TCR β chains via the immunoSEQ® assay at survey (tissue) or deep (blood) resolution. TCR metrics data, as defined in the Analyzer Export Guide (https://www.adaptivebiotech.com/wp-content/uploads/2019/07/MRK-00342_immunoSEQ_TechNote_DataExport_WEB_REV.pdf), were downloaded from the immunoSEQ® portal (Adaptive Biotechnologies). The two-sample Kolmogorov–Smirnov test was used to calculate the statistical significance of differences in TCR metrics by BOR. Cox proportional-hazards model analysis was run and forest plots were generated with the finalfit 1.0.3 package (https://github.com/ewenharrison/finalfit) in R version 4.1.0 [8]. The hazard ratio (HR), 95% confidence interval (CI), and *P* values were reported for each association of TCR metric with PFS.

#### RNA sequencing and whole exome sequencing (WES)

RNA sequencing was performed by NeoGenomics Laboratories (Fort Myers, FL, USA) using the Illumina Stranded Total RNA preparation. Baseline FFPE tumor tissue genomic DNA from three patients was sequenced at NeoGenomics Laboratories using the xGen Prism DNA Library Prep Kit and the IDT xGen Exome Research Panel V2 (both Integrated DNA Technologies, Coralville, IA, USA). The detailed RNA and WES sequencing methods are described in Online Resource 1 (Supporting Document S1).

## Results

### Patients

The disposition of patients is shown in Fig. S1 (Online Resource 1). A total of 14 patients were enrolled, of whom 4 (29%) were screening failures. Ten patients received imaradenant treatment (50-mg QD cohort, *n* = 3; 75-mg QD cohort, *n* = 7). All ten patients ultimately discontinued imaradenant treatment; the reasons for discontinuation were worsening of the patient’s general condition (eight patients, 80%), subjective disease progression (one patient, 10%), and patient decision (one patient, 10%). All ten patients were included in the safety analysis set, the PK analysis set, and the tumor response analysis set. Nine patients were included in the DLT analysis set, with one patient being excluded due to not receiving at least 75% of the imaradenant dose during Cycle 0 and Cycle 1.

The baseline demographic and clinical characteristics of the study patients are summarized in Table 1. Overall, the median age of patients was 65.5 (range, 49–80) years, the majority were male (nine patients, 90%), and all were Asian. The ECOG performance status for the majority of patients (eight patients, 80%) was 0. All patients had received at least one course of prior therapy; the majority of patients (nine patients, 90%) had received three or more courses of prior chemotherapy regimens. There were no major differences between the treatment cohorts in demographic and clinical characteristics. Other disease characteristics, such as the American Joint Committee on Cancer disease, tumor, node, and metastasis stages, all differed among patients (Table S2 in Online Resource 1).

**Table 1:** Baseline demographic and clinical characteristics

Characteristic		Imaradenant 50-mg QD (*n* = 3)	Imaradenant 75-mg QD (*n* = 7)	Total (*N* = 10)
Age (years)	Median (min–max)	68.0 (63–77)	63.0 (49–80)	65.5 (49–80)
Age group, *n* (%)	20–64 years	1 (33)	4 (57)	5 (50)
	≥ 65 years	2 (67)	3 (43)	5 (50)
Sex, *n* (%)	Male	2 (67)	7 (100)	9 (90)
	Female	1 (33)	0	1 (10)
Race, *n* (%)	Asian	3 (100)	7 (100)	10 (100)
Height (cm)	Median (min–max)	160.0 (154.0–170.0)	162.0 (150.0–168.0)	161.5 (150.0–170.0)
Weight (kg)	Median (min–max)	58.0 (57.0–71.0)	70.0 (58.0–81.0)	68.5 (57.0–81.0)
ECOG PS, *n* (%)	0	3 (100)	5 (71)	8 (80)
	1	0	2 (29)	2 (20)
Primary tumor location	Kidney	1 (33)	0	1 (10)
	Prostate gland	1 (33)	5 (71)	6 (60)
	Urethra	0	1 (14)	1 (10)
	Uterus	1 (33)	0	1 (10)
	Other	0	1 (14)	1 (10)
Histology type	Adenocarcinoma	1 (33)	4 (57)	5 (50)
	Adenocarcinoma (NOS)	0	2 (29)	2 (20)
	Clear cell carcinoma	1 (33)	0	1 (10)
	Leiomyosarcoma	1 (33)	1 (14)	2 (20)
Received ≥ 1 course of prior therapy, *n* (%)	Systemic therapy	3 (100)	7 (100)	10 (100)
	Radiation	1 (33)	3 (43)	4 (40)
	Surgery	3 (100)	2 (29)	5 (50)
Prior chemotherapy regimens, *n* (%)	2	0	1 (14)	1 (10)
	3 +	3 (100)	6 (86)	9 (90)

*ECOG PS* Eastern Cooperative Oncology Group performance status, *NOS* not otherwise specified, *QD* once daily

### Safety

The duration of exposure of imaradenant is summarized in Table S3 (Online Resource 1). The total median (range) treatment duration was similar between the two cohorts: 2.10 (1.1–3.5) months for the 50-mg QD cohort and 2.14 months for the 75-mg QD cohort. Actual median treatment duration was slightly shorter for the 75-mg QD cohort (1.94 [0.3–4.8] months), indicating some dose interruptions. No patient experienced a dose reduction, but four patients (57%) in the imaradenant 75-mg QD cohort experienced a dose interruption during the study. The mean (SD) relative dose intensity was 100% (0%) for patients in the 50-mg QD cohort and 91% (16%) for patients in the 75-mg QD cohort.

AEs are summarized in Table 2. Overall, nine patients (90%) experienced AEs during the study and five (50%) reported AEs that were considered causally related to the study treatment by the investigator. No patient experienced an AE of CTCAE Grade ≥ 3, and no deaths or SAEs were reported during the study. There were no discontinuations of study treatment due to AEs; however, two patients (29%) in the imaradenant 75-mg QD cohort experienced AEs (malaise and nausea, and influenza) leading to dose interruptions of the study treatment. The AEs causally related to study treatment by SOC and PT and by CTCAE grade are also summarized in Table 2. All AEs recorded were Grades 1 or 2, and no Grade ≥ 3 AEs were reported. Of the five patients with causally related AEs, three from the imaradenant 75-mg cohort had Grade 2 AEs of nausea, diarrhea, and/or malaise. No DLTs were reported during the study. Although some patients presented with hematological parameters, clinical chemistry parameters, and urinalysis parameters that were lower or higher than baseline during the study, no trends or differences between treatment groups were observed. No clinically important changes were observed in either vital signs or ECG values.

**Table 2:** Summary of AEs (safety analysis set)

	Maximum reported CTCAE grade^b^	Number (%) of patients^a^		
		Imaradenant 50-mg QD (*n* = 3)	Imaradenant 75-mg QD (*n* = 7)	Total (*N* = 10)
Any AE	Any grade	2 (67)	7 (100)	9 (90)
	Grade 1–2	2 (67)	7 (100)	9 (90)
	Grade ≥ 3	0	0	0
Any AE leading to dose interruption of treatment	Any grade	0	2 (29)	2 (20)
Any AE causally related to treatment^c^	Any grade	1 (33)	4 (57)	5 (50)
	Grade 1	1 (33)	1 (14)	2 (20)
	Grade 2	0	3 (43)	3 (30)
Treatment-related AEs, System organ class, Preferred term^d^				
Metabolism and nutrition disorders	Grade 1	0	1 (14)	1 (10)
Decreased appetite	Grade 1	0	1 (14)	1 (10)
Psychiatric disorders	Grade 1	0	1 (14)	1 (10)
Insomnia	Grade 1	0	1 (14)	1 (10)
Vascular disorders	Grade 1	0	1 (14)	1 (10)
Orthostatic hypotension	Grade 1	0	1 (14)	1 (10)
Gastrointestinal disorders	Grade 1	1 (33)	0	1 (10)
	Grade 2	0	3 (43)	3 (30)
Diarrhea	Grade 2	0	1 (14)	1 (10)
Gastritis	Grade 1	1 (33)	0	1 (10)
Nausea	Grade 1	1 (33)	1 (14)	2 (20)
	Grade 2	0	2 (29)	2 (20)
Vomiting	Grade 1	1 (33)	1 (14)	2 (20)
General disorders and administration site conditions	Grade 1	1 (33)	1 (14)	2 (20)
	Grade 2	0	1 (14)	1 (10)
Malaise	Grade 1	0	1 (14)	1 (10)
	Grade 2	0	1 (14)	1 (10)
Pyrexia	Grade 1	1 (33)	0	1 (10)

*AE* adverse event, *CTCAE* Common Terminology Criteria for Adverse Events version 4.03, *QD* once daily

^a^Patients with multiple events in the same category were counted only once in that category. Patients with events in more than one category were counted once in each of those categories. Includes AEs with an onset date on or after the date of first dose and up to and including 30 days following the date of last dose of study medication

^b^The maximum CTCAE grade was reported for each event occurring per patient

^c^As assessed by the investigator and programmatically derived from causality assessments

^d^Classified using the Medical Dictionary for Regulatory Activities version 23.1

One patient experienced anemia during the follow-up; this event was not causally related to study treatment and was reported as not recovered/not resolved. No AEs related to clinical chemistry, urinalysis, or ECG were reported. AEs related to vital signs were orthostatic hypotension and weight decrease; these events were also not causally related to the study treatment and were reported as not recovered/not resolved.

### PK analysis

**Fig. 1:**
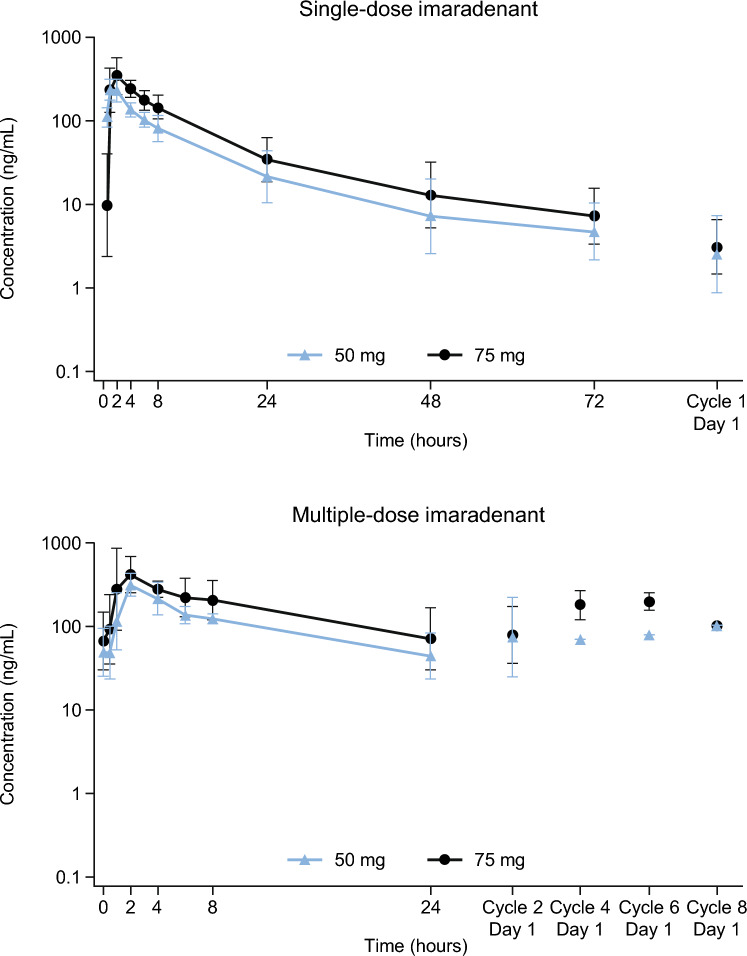
Geometric mean ± SD plasma concentration (ng/mL) of single-dose and multiple-dose 50-mg and 75-mg imaradenant over time (log scale, *n* = 3 for 50 mg and *n* = 7 for 75 mg, pharmacokinetics analysis set)

The geometric mean ± SD plasma concentrations of imaradenant and its metabolites over time after 50-mg and 75-mg dosing are shown in Fig. 1, and Supporting Figs. S2 and S3 (Online Resource 1). The plasma concentration of imaradenant and its metabolites at pre-dose on Cycle 4 Day 1 for two patients (both receiving 50 mg) were excluded from the analysis due to a drug holiday that occurred before Cycle 4 Day 1. In addition, an error of plasma concentration of the inactive metabolite of imaradenant (SSP-005173X) in the samples collected at 6 h post-dose on Cycle 1 Day 15 from one patient was identified after database lock. Thus, these data were excluded from the PK analysis. The PK parameters of imaradenant and its metabolites following single and multiple oral administration of imaradenant are summarized in Tables S4, S5, and S6 (Online Resource 1). Imaradenant was rapidly absorbed after single or multiple oral administration with median times of maximum observed concentrations sampled during the dosing interval (t_max_) of 1.08 h (50 mg; range, 0.95–1.95) and 2.00 h (75 mg; range, 0.92–5.52 h) (Table S4 in Online Resource 1). Following t_max_, levels of imaradenant declined in a biphasic manner with mean (SD) t_½_ values of 16.3 (7.1) and 18.3 (6.2) h following single-dose administrations of 50 and 75 mg, respectively (Table S4 in Online Resource 1). After multiple dosing, there was very little accumulation in imaradenant exposure, with geometric mean accumulation ratios of maximum concentration (C_max_) of 1.3 (coefficient of variation [CV]% 16.1) for 50-mg QD to 1.4 (CV% 59.0) for 75-mg QD and area under the concentration–time curve (AUC)_0–24_ of 1.4 (CV% 5.5) for 50-mg QD to 1.5 (CV% 23.1) for 75-mg QD (Table S4 in Online Resource 1). There was no marked time dependency with geometric mean temporary change parameters of 1.1 (CV% 17.4) for imaradenant 50-mg QD and 1.1 (CV% 18.4) for 75-mg QD (Table S4 in Online Resource 1). There was a dose-proportional increase of C_max_ and AUC between the two imaradenant dose levels, but also a moderate inter-subject variability in C_max_ and AUC, with geometric mean CV% values ranging from 29.0% to 66.8%, leading to the overlapping exposures observed between dose levels (Table S4 in Online Resource 1). The geometric mean metabolite to parent ratios of SSP-005174X (active) and SSP-005173X (inactive) were 8.6%–14.9% and 1.8%–6.6%, respectively. Dose-normalized C_max_, AUC_0–t_, C_max,ss_, and AUC_ss_ of imaradenant versus dose are shown in Fig. S4 (Online Resource 1).

### Tumor response

The BOR is shown in Table 3; none of the ten patients in either imaradenant cohort showed a response. Of the non-responders, the BOR was stable disease for ≥ 9 weeks in three (30%) patients overall (one patient in the imaradenant 50-mg QD cohort [33%] and two in the 75-mg QD cohort [29%]). Most patients showed RECIST progression (six patients overall [60%]; two in the imaradenant 50-mg QD cohort [67%], and four in the 75-mg QD cohort [57%]). As no patient achieved disease control at Week 15, the DCR was 0%. Furthermore, no patients with prostate cancer showed any prostate-specific antigen response. The median percentage changes from baseline in target lesion size were 20.92% (range, 20.0%–21.8%) in the imaradenant 50-mg QD cohort and 17.95% (range, −15.8%–21.3%) in the 75-mg QD cohort.

**Table 3:** Best objective response (tumor response analysis set)

Best objective response	Number (%) of patients		
	Imaradenant 50-mg QD (*n* = 3)	Imaradenant 75-mg QD (*n* = 7)	Total (*N* = 10)
CR/PR	0	0	0
Non-response, total	3 (100)	7 (100)	10 (100)
Stable disease ≥ 9 weeks	1 (33)	2 (29)	3 (30)
PD	2 (67)	4 (57)	6 (60)
RECIST progression	2 (67)	4 (57)	6 (60)
Death	0	0	0
Not evaluable	0	1 (14)	1 (10)
Stable disease < 9 weeks	0	0	0
Incomplete post-baseline assessments	0	1 (14)	1 (10)
Patients with response [95% CI]	0 [0.0, 70.8]	0 [0.0, 41.0]	0 [0.0, 30.8]

*CI* confidence interval, *CR* complete response, *PD* progressive disease, *PR* partial response, *QD* once daily; *RECIST* Response Evaluation Criteria in Solid Tumors

**Fig. 2:**
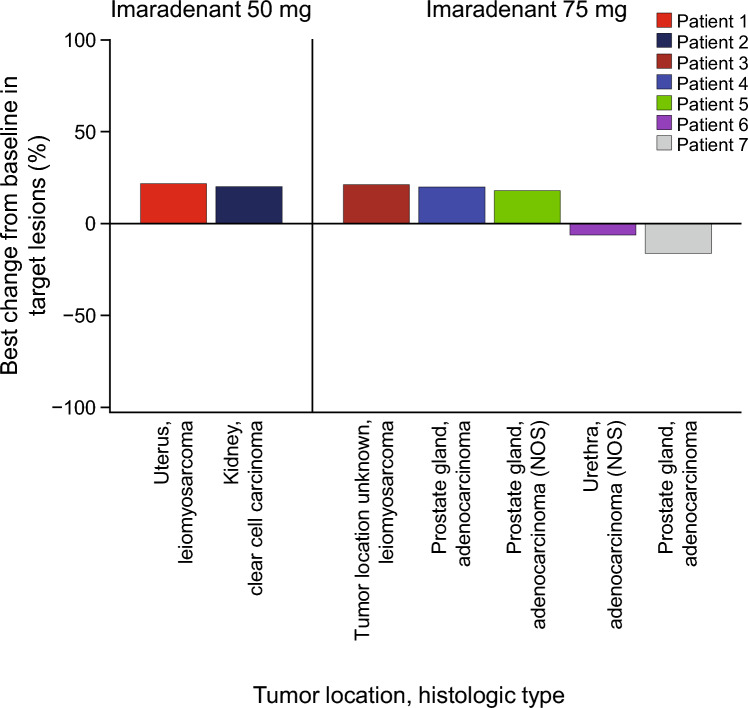
Percentage change in target lesion size from baseline in patients treated with imaradenant 50 mg or 75 mg once daily. Tumor location information was not available for patient 3. *NOS* not otherwise specified

Among the seven patients with target lesion size data, two patients in the imaradenant 75-mg QD cohort showed a reduction in target lesion size at 8 weeks (Fig. 2); however, this was not maintained over time. In one patient, the primary tumor location was the urethra and the histology type was adenocarcinoma (not otherwise specified), and in the other patient, the primary tumor location was the prostate gland and histology type was adenocarcinoma.

### ctDNA analysis

**Fig. 3:**
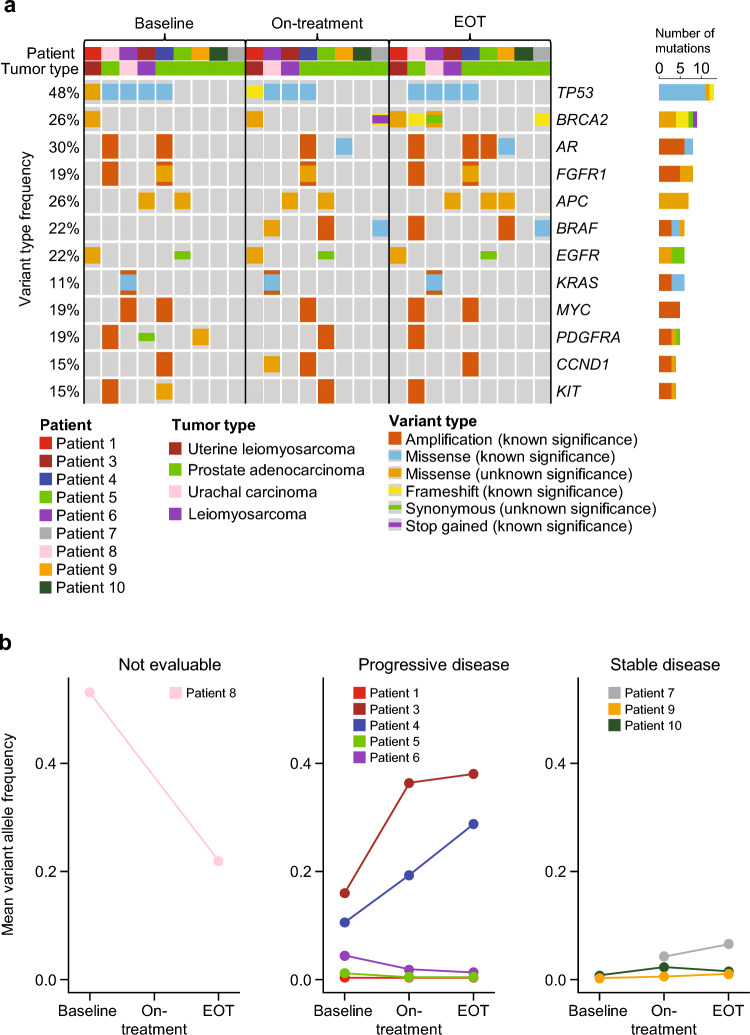
Mean variant allele frequency by clinical response group over time. **a** Genetic variant type and frequency by patient and tumor type over time. **b** Mean variant allele frequency by clinical response group over time. On-treatment samples were taken at Cycle 3/Day 1. No on-treatment sample was available for patient 8. *EOT* end of treatment

All 28 plasma samples across three time points (baseline, Cycle 3/Day 1 [on-treatment], and end of treatment) from ten unique patient samples were successfully processed for ctDNA analysis. In total, 25/28 samples (89%) from 9/10 patients assessed showed somatic mutations (after excluding common putative clonal hematopoiesis variants) (Fig. 3a). The most common somatically mutated genes in the sample set were tumor protein p53 (*TP53*), androgen receptor (*AR*), adenomatous polyposis coli (*APC*), and breast cancer type 2 susceptibility protein (*BRCA2*)*. AR* alterations (amplifications and ligand-binding domain [LBD] variant T878A) were found in 4/9 (44%) patients. Of patients with prostate cancer whose ctDNA data were available, 4/6 (67%) had *AR* alterations (one single nucleotide variant and three copy number variants). Serine/threonine-protein kinase B-Raf (*BRAF*) alterations (amplifications and kinase-domain variant K601E) were found in 4/9 (44%) patients. VAF, a marker of tumor mutation burden, showed no obvious longitudinal post-treatment trend across response groups. Two of five patients (40%) with PD showed an increase in VAF by the end of treatment (Fig. 3b).

### TCR repertoire analysis

FFPE baseline tumor samples from six patients and baseline blood samples from five patients were evaluated for their TCR repertoire. The lack of on-treatment samples prevented the evaluation of changes in the peripheral repertoire with imaradenant treatment. There was no significant association of baseline tumoral or peripheral TCR repertoire clonality (Fig. S5 in Online Resource 1) or diversity metrics with BOR (stable disease, PD) or PFS.

## Discussion

In this phase I, open-label study of imaradenant 50- and 75-mg QD in ten Japanese patients with advanced solid malignancies, we found that imaradenant demonstrated an acceptable safety profile and resulted in a BOR of stable disease ≥ 9 weeks for 30% of patients. All patients in this study eventually discontinued treatment, and the reasons for discontinuation were mainly worsening of their general condition; no patients discontinued because of AEs or SAEs. To avoid unnecessarily high exposure to imaradenant and potential treatment-related AEs, we prespecified a maximum dose of 75-mg QD, which was consistent with previous research in an overseas study [5]. The maximum tolerated dose of imaradenant was not reached in this study as no DLTs were reported.

The observed safety and clinical laboratory assessments were in line with the observed tolerability profile of imaradenant (AstraZeneca, data on file). Overall, nine (90%) patients reported AEs. No AE with an outcome of death, SAEs, or AEs leading to discontinuation were reported. No AEs of CTCAE Grade ≥ 3 were reported. The most common AEs reported were nausea, malaise, decreased appetite, and vomiting. This is consistent with the previous phase Ia/b trial that reported diarrhea, nausea, fatigue, dizziness, decreased appetite, and vomiting as common treatment-related AEs [5]. Nausea and vomiting had previously been considered a potential risk for imaradenant based on the available data from other agents (A_2A_R antagonists) with related mechanisms of action [9–11]. Data from other studies support this claim, with nausea and vomiting among the most commonly observed AEs following imaradenant treatment (AstraZeneca, data on file). In the present study, two (29%) patients in the 75-mg QD cohort reported AEs (malaise and nausea, and influenza), leading to dose interruptions of the study treatment. Based on a review of all currently available information, it was deemed that there is a reasonable possibility of a causal relationship between imaradenant and nausea and vomiting, suggesting that pretreatment with anti-emetics would be appropriate.

In the PK analyses, we observed that imaradenant was rapidly absorbed after single or multiple oral administrations with a median t_max_ of 1.1–2.0 h. Following t_max_, plasma concentrations of imaradenant declined in a bi- or triphasic manner following a single-dose administration. After multiple dosing, little accumulation in exposure was observed. A dose-proportional increase of C_max_ and AUC was observed between the two dose levels (50 mg vs 75 mg). A moderate inter-patient variability in C_max_ and AUC was shown, leading to the overlapping exposures observed between these dose levels.

No firm conclusions regarding clinical efficacy can be made from the findings of this study, as the data collected are preliminary and the sample size was small. However, we did observe an overall stable disease rate of 30% and a reduction in lesion size in two patients receiving the 75-mg QD dose.

Most patients with prostate cancer (4/6) had alterations in *AR*, including copy number changes and a T878A variant of the LBD. This was not unexpected, as all six patients with prostate cancer had disease progression on prior abiraterone treatment, and *AR* amplifications and point mutations in LBD are both associated with anti-androgen therapy resistance [12–15].

Somatic mutations detected in ctDNA from plasma may signal disease progression and indicate the response to therapies [16]. Two patients (40%) in our study experienced both an increase in VAF and disease progression, suggesting that analyzing ctDNA dynamics may indicate disease progression through minimally invasive technology. Although the TCR repertoire was expected to be a predictive biomarker of response to immuno-oncology medicine [17], we did not observe any significant association of baseline tumoral or peripheral TCR repertoire clonality or diversity metrics with tumor response because of the lack of on-treatment samples. Additionally, while we evaluated the association between clinical response and baseline T cell-inflamed, adenosine-relevant gene expression signatures in the tumors, we found no significant association owing to the limited data available in this study. However, given our current understanding of the T cell and adenosine pathway biology, we recommend the continued assessment of these parameters in studies targeting the PD-1 axis and adenosine pathway [2, 18–20].

We acknowledge the limitations of this study. The small sample size reduced the power of statistical analyses of the TCR sequencing. Additionally, as none of the patients in this study responded to imaradenant treatment, we were unable to associate the biomarker data with clinical responses. Moreover, we were not able to evaluate the pharmacodynamics of the A_2A_R inhibition because of the absence of matched on-treatment patient samples for WES, RNA sequencing, and TCR sequencing.

In conclusion, imaradenant 50- and 75-mg QD demonstrated an acceptable safety profile and was generally well tolerated by the population of Japanese patients with advanced solid malignancies in this study, with no new or unexpected safety concerns. Combining imaradenant with immunotherapy may decrease immune suppression in the TME, thereby increasing the efficacy of immunotherapy.

### Supplementary Information

Below is the link to the electronic supplementary material.

Supplementary file1 (PDF 404 KB)

## Data Availability

Data underlying the findings described in this manuscript may be obtained in accordance with AstraZeneca’s data sharing policy described at https://astrazenecagrouptrials.pharmacm.com/ST/Submission/Disclosure. Data for studies directly listed on Vivli can be requested through Vivli at www.vivli.org. Data for studies not listed on Vivli can be requested through Vivli at https://vivli.org/members/enquiries-about-studies-not-listed-on-the-vivli-platform/. The AstraZeneca Vivli member page is also available outlining further details: https://vivli.org/ourmember/astrazeneca/.
