# Supplementary material for: Safety and pharmacokinetics of imaradenant (AZD4635) in Japanese patients with advanced solid malignancies: a phase I, open-label study
Source: Cancer Chemother Pharmacol. 2023 Dec 13;93(4):341–52. doi: 10.1007/s00280-023-04605-9 (PMC10950984; doi:10.1007/s00280-023-04605-9)
Supplement: Supplementary file 1 — Supplementary file1 (PDF 404 KB) [file 280_2023_4605_MOESM1_ESM.pdf]

Online Resource 1

Safety and pharmacokinetics of imaradenant (AZD4635) in Japanese patients with advanced solid malignancies: a phase I, open-label study

Cancer Chemotherapy and Pharmacology

Nobuaki Matsubara,* Shota Kusuhara, Noboru Yamamoto, Kazuki Sudo, Masahiko Yanagita, Kosho Murayama, Hisashi Kawasumi, Deanna L. Russell, Da Yin, Toshio Shimizu

*Correspondence:

Nobuaki Matsubara

Department of Medical Oncology, National Cancer Center Hospital East, 6-5-1 Kashiwanoha, Kashiwa, Chiba 277-8577, Japan

Tel: +81-4-7133-1111

Fax: +81-4-7134-6922

E-mail: nmatsuba@east.ncc.go.jp

Supporting Document S1 RNA and whole exome sequencing (WES) sequencing

RNA sequencing methods

RNA sequencing was performed by NeoGenomics Laboratories (Fort Myers, FL, USA) using the Illumina Stranded Total RNA preparation. The RNAseq pipeline implemented in bcbio-nextgen version 1.2.7 (https://github.com/bcbio/bcbio-nextgen) was used for quality control and gene expression quantification. Reads were aligned to the GRCh38 *Homo sapiens* genome (University of California Santa Cruz Genomics Institute, Santa Cruz, CA, USA), augmented with transcript information from Ensembl release 86 using STAR's 2-pass mapping mode (version 2.6.1d; https://github.com/alexdobin/STAR). Alignments were evaluated for evenness of coverage, rRNA content, genomic context of alignments, and complexity using a combination of FastQC (Babraham Bioinformatics, Cambridge, UK), Qualimap [1], and custom tools. Transcripts per million (TPM) measurements per isoform were generated by alignment-based quantification using Salmon (version 1.4.0; https://github.com/COMBINE-lab/salmon) and used to estimate the abundance of genes. R version 4.1.0 was used for all statistical computing downstream [2]. Gene set variation analysis 1.40.1 [3] (gsva function, 'gsva' method) was performed on the TPM normalized data to calculate the signature score of each sample based on AZsigDB, which is AstraZeneca's curated database of annotated gene sets, similar to MSigDB [4]. Mean and median signature scores were calculated based on the average and median expression across all genes comprising the gene expression signature.

Whole exome sequencing (WES) methods

Baseline FFPE tumor tissues from three patients were sequenced at NeoGenomics Laboratories using the xGen Prism DNA Library Prep Kit and the IDT xGen Exome Research Panel V2 (both Integrated DNA Technologies, Coralville, IA, USA). Sequencing data were demultiplexed and passed through a bcl-to-fastq conversion program (bcl2fastq v2.20.0.422; Illumina, San Diego, CA, USA). Fastq files were analyzed using pipeline software bcbio-nextgen. Reads were aligned to the hg38 reference using bwa mem v0.7.17 (https://github.com/lh3/bwa), and sequencing duplicates for each unique molecular identifier were collapsed into a single consensus read using fgbio v1.0.0 (Fulcrum Genomics, Boulder, CO, USA). All software was run using best practice parameters established within the bcbio workflow or in-house. Variant calling was performed using VarDict v1.7.0 (AstraZeneca) [5], down to a VAF of 1% (before filtering and curation) and variant effects annotated by snpEff v4.3.1t [6]. Filtering of

non-cancer variants (i.e., common polymorphisms) was performed per VarDict best practice. For downstream analysis, our filtering criteria removed (1) potential germline and CHIP mutations, (2) variants of unknown significance and potential defects (variant/total depth < 3), and (3) variants seen in > 40% of samples. If the samples were not homogeneous, but came from a single sequencer run, we expected recurring variants to be caused by sequencing artifacts. In addition, common mutations were expected to be germline. The post-processing pipeline calculated the number and the percentage of samples harboring each variant. High percentage variants were potentially either artifacts or germline variants. Single nucleotide variants and insertion/deletion variants were called by VarDict, while copy number variants were called by Seq2c (https://github.com/AstraZeneca-NGS/Seq2C). The most frequent variants were reported on Oncoprint plot using the ComplexHeatmap 2.8.0 package [7].

RNA and WES sequencing results

Baseline FFPE tumor tissue samples from three patients were evaluated by RNA and WES sequencing; because of the small sample size, differential gene expression analysis could not be performed. Five gene expression signatures of interest, including adenosine signaling, were scored on the three patient baseline tumors (**Fig. S6**). Twenty-six genes with alterations were found in at least 2/3 patients, including *BRAF* and checkpoint kinase 2 (*CHEK2*) each in 3/3 patients and *TP53* in 2/3 patients. None of the three patients in this dataset carried a mutated *AR* gene.

References:

1. García-Alcalde F, Okonechnikov K, Carbonell J et al (2012) Qualimap: evaluating next-generation sequencing alignment data. Bioinformatics 28:2678–2679. https://doi.org/10.1093/bioinformatics/bts503
2. R Core Team (2021) R: A language and environment for statistical computing. R Foundation for Statistical Computing, Vienna, Austria. https://www.R-project.org/
3. Hänzelmann S, Castelo R, Guinney J (2013) GSVA: gene set variation analysis for microarray and RNA-seq data. BMC Bioinformatics 14:7. https://doi.org/10.1186/1471-2105-14-7
4. Subramanian A, Tamayo P, Mootha VK et al (2005) Gene set enrichment analysis: a knowledge-based approach for interpreting genome-wide expression profiles. Proc Natl Acad Sci U S A 102:15545–15550. https://doi.org/10.1073/pnas.0506580102

5. Lai Z, Markovets A, Ahdesmaki M et al (2016) VarDict: a novel and versatile variant caller for next-generation sequencing in cancer research. Nucleic Acids Res 44:e108. https://doi.org/10.1093/nar/gkw227
6. Cingolani P, Platts A, Wang le L et al (2012) A program for annotating and predicting the effects of single nucleotide polymorphisms, SnpEff: SNPs in the genome of Drosophila melanogaster strain w1118; iso-2; iso-3. Fly (Austin) 6:80–92. https:/doi.org/10.4161/fly.19695
7. Gu Z, Eils R, Schlesner M (2016) Complex heatmaps reveal patterns and correlations in multidimensional genomic data. Bioinformatics 32:2847–2849. https://doi.org/10.1093/bioinformatics/btw313

Supporting Figures

Supporting Fig. S1 Patient disposition

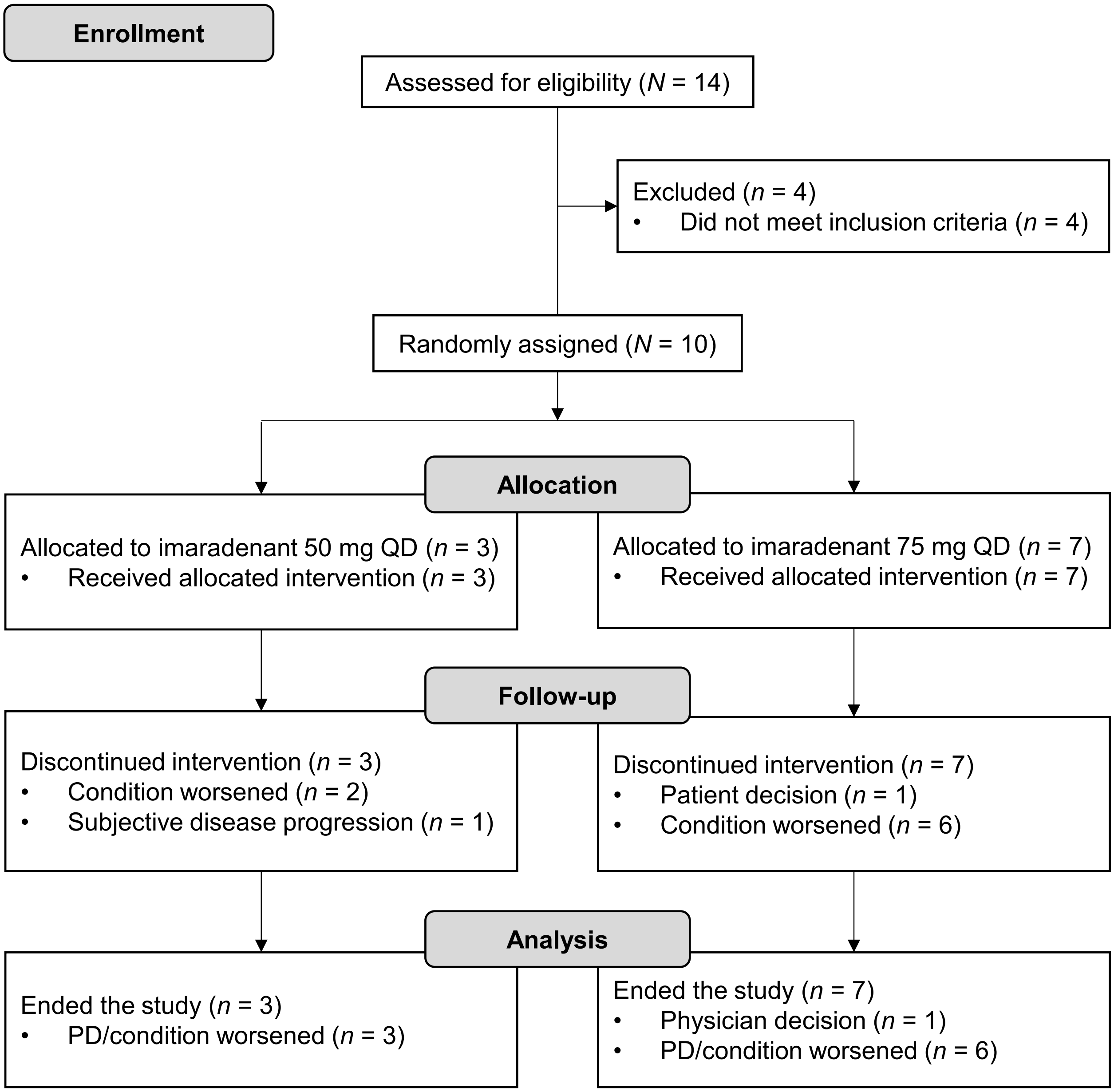

Abbreviations: QD, once daily; PD, progressive disease.

Supporting Fig. S2 Geometric mean ± SD plasma concentrations (ng/mL) of the active metabolite SSP-005174X over time for patients receiving either 50 mg or 75 mg imaradenant (log scale, *n* = 3 for 50 mg and *n* = 7 for 75 mg, pharmacokinetics analysis set)

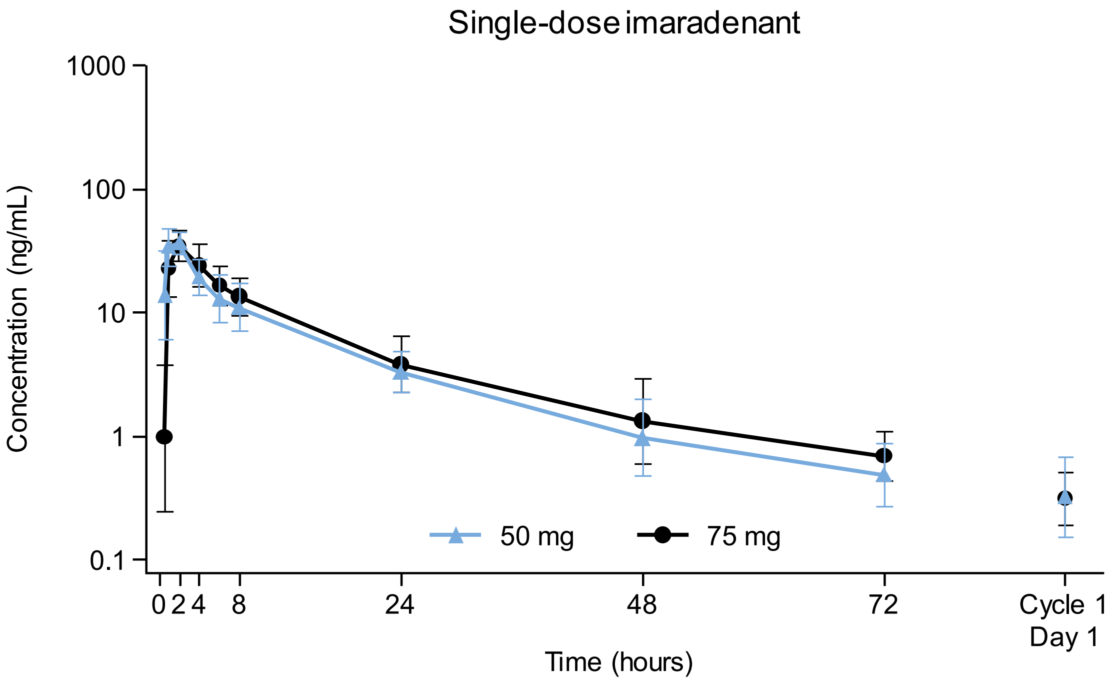

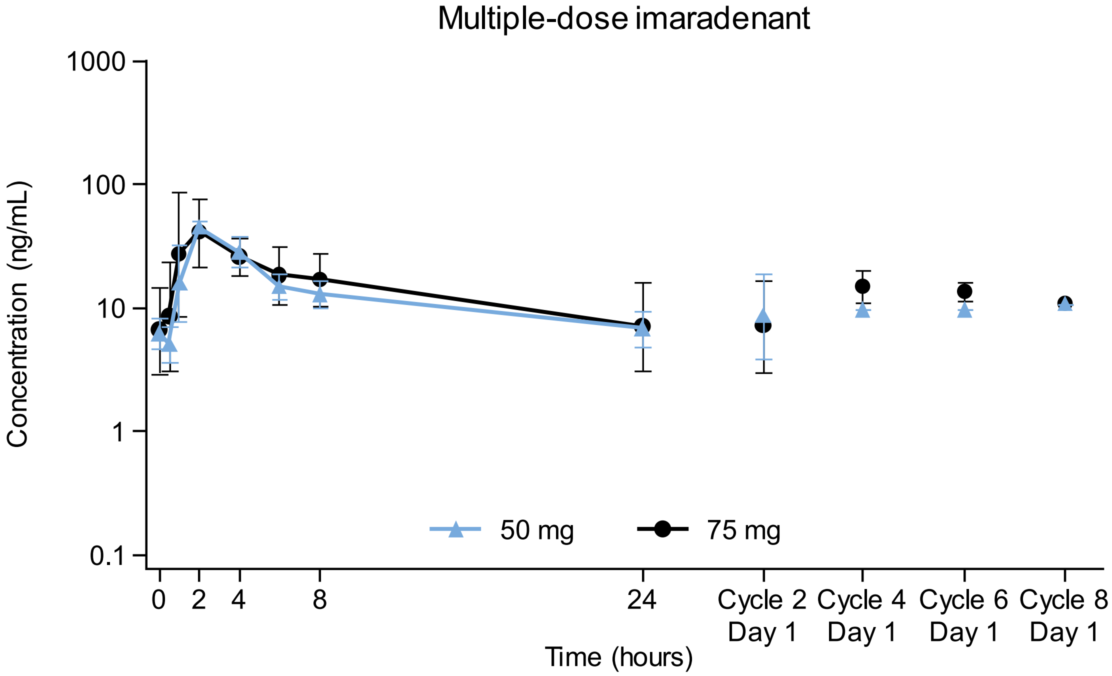

Supporting Fig. S3 Geometric mean ± SD plasma concentrations (ng/mL) of the inactive metabolite SSP-005173X over time for patients receiving either 50 mg or 75 mg imaradenant (log scale, *n* = 3 for 50 mg and *n* = 7 for 75 mg, pharmacokinetics analysis set)

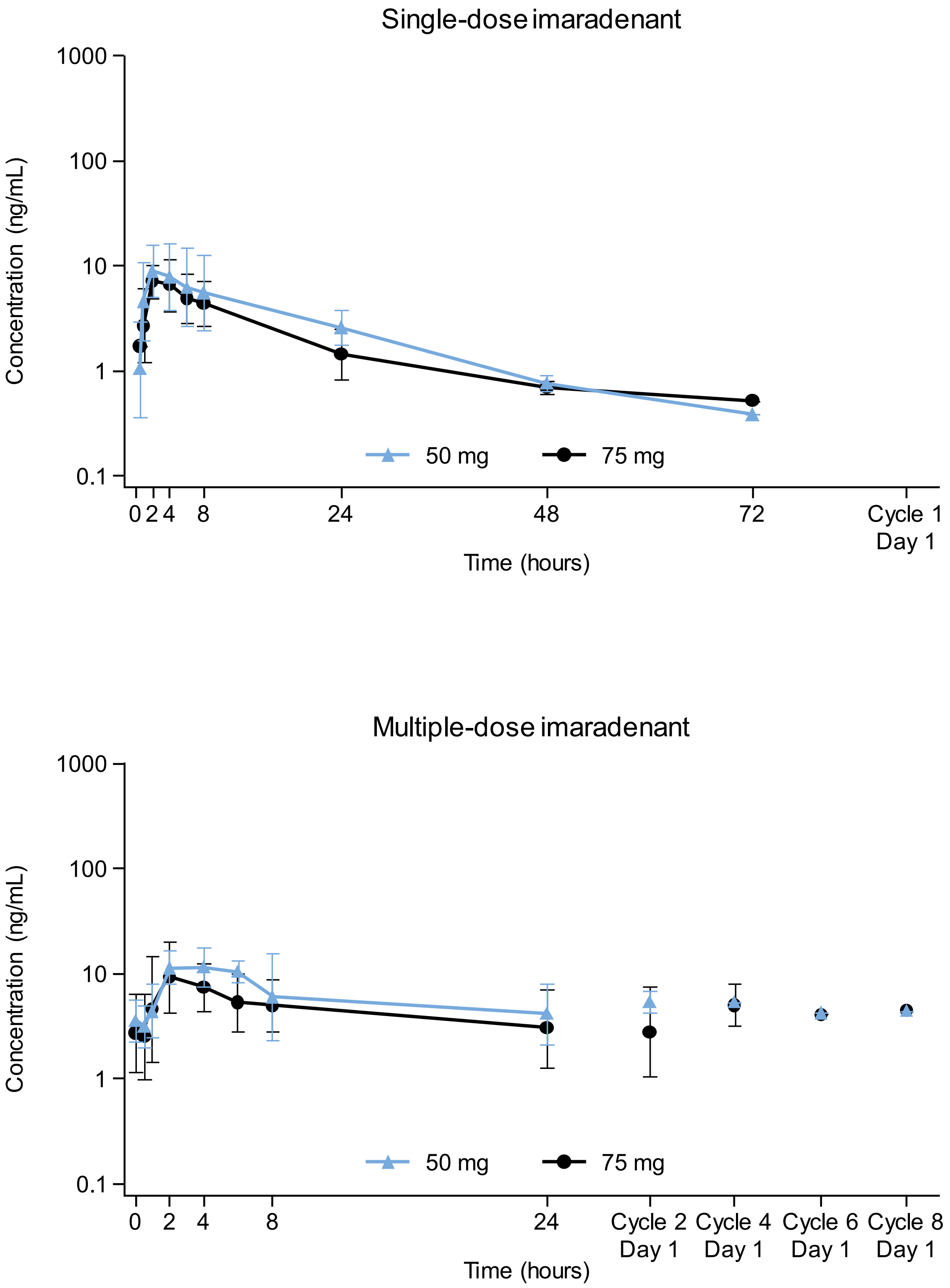

Supporting Fig. S4 Dose-normalized C_{max} (A), $AUC_{0–t}$ (B), $C_{max,ss}$ (C), and $AUC_{0–24,ss}$ (D) of imaradenant versus dose

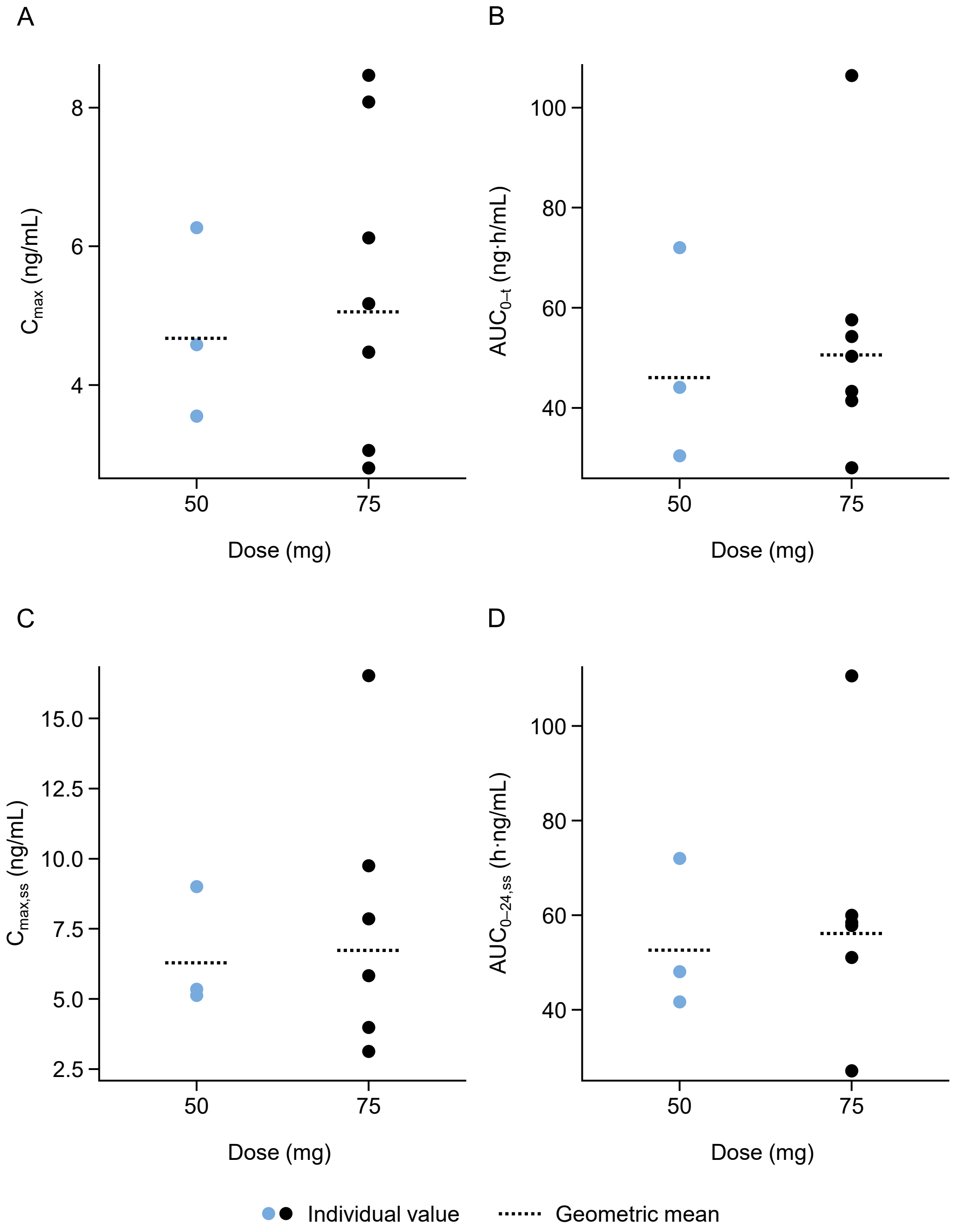

Abbreviations: AUC, area under the concentration–time curve; $AUC_{0–t}$, AUC up to the last measurable concentration; $AUC_{0–24}$, AUC from time 0 to 24 h; C_{max}, maximum observed concentration sampled during the dosing interval; SS, steady state.

Supporting Fig. S5 T cell receptor repertoire analysis of baseline tumor and blood samples

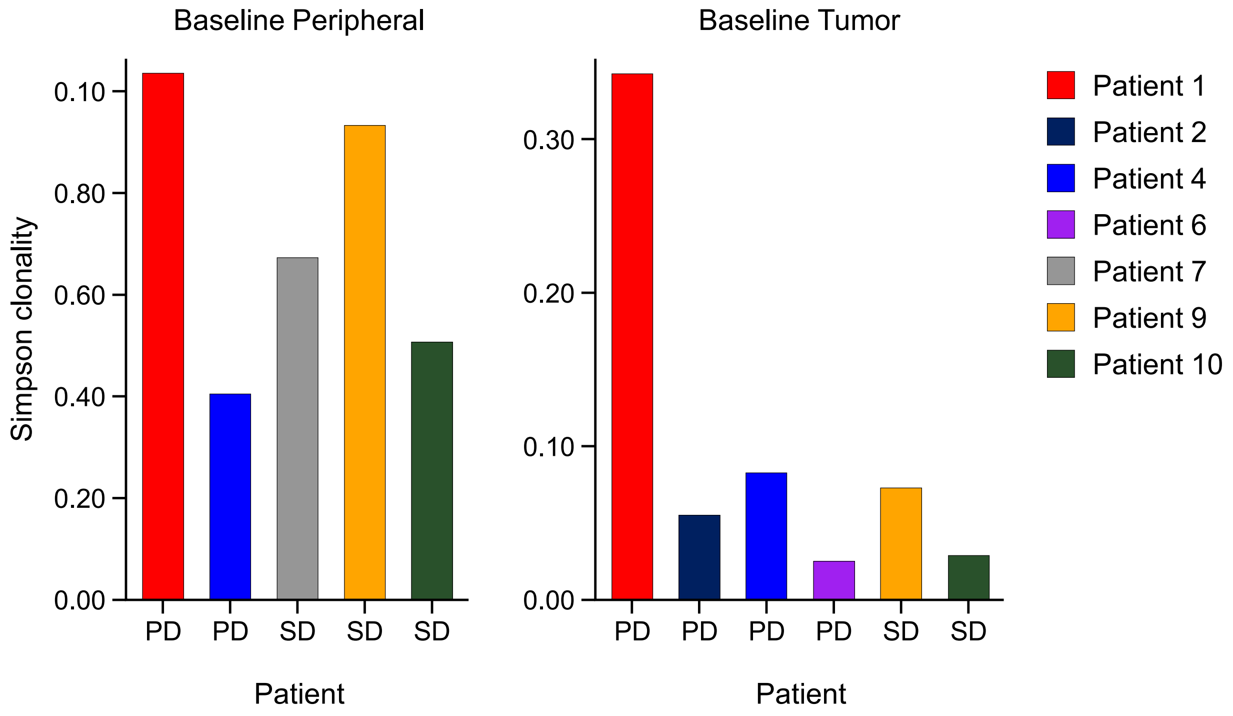

Abbreviations: PD, progressive disease; SD, stable disease.

Supporting Fig. S6 Select gene expression signatures reflecting intra-tumoral adenosine levels and pre-existing immune status in baseline tumors from three patients

Abbreviations: IFN, interferon; NK, natural killer cells.

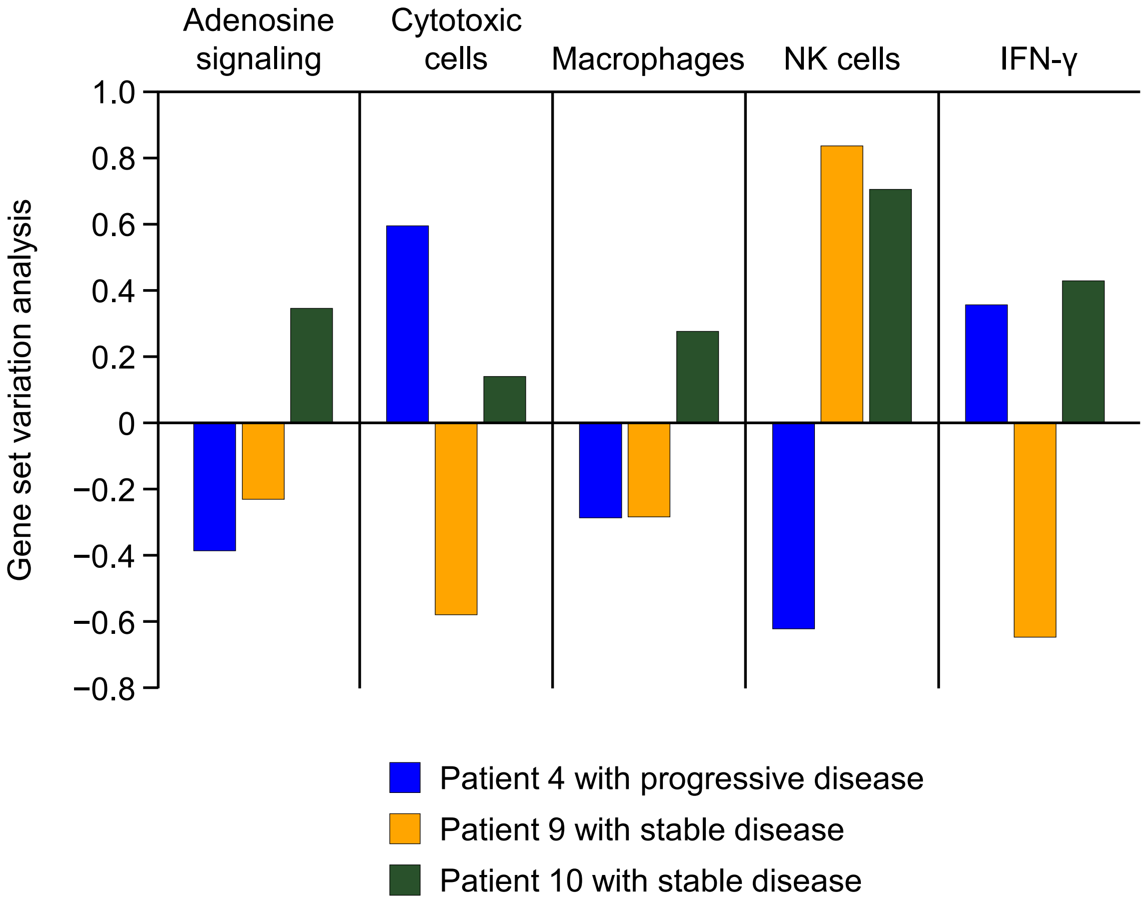

Adenosine signaling signature genes: *PPARG, CYBB, COL3A1, FOXP3, LAG3, APP, CD81, GPI, PTGS2, CASP1, FOS, MAPK1, MAPK3, CREB1*

Cytotoxic cells signature genes: *CTSW, GNLY, GZMA, GZMB, GZMH, KLRB1, KLRD1, KLRK1, PRF1, NKG7*

Macrophage signature genes: *CD163, CD68, CD84, MS4A4A*

NK cells signature genes*: NCR1, XCL2, XCL1*

IFN-γ signature genes*: LAG3, CXCL9, IFNG, CD274*

Supporting Tables

Supporting Table S1 Study design

Cohort	Imaradenant once daily	Patients
1	50 mg	At least 3[a, b]
2	75 mg	At least 6[b]

[a]If no dose-limiting toxicities were observed with the first 3 patients in a cohort, the tolerability assessment would be done with 3 evaluable patients.

[b]The cohort could be expanded to include a maximum of 12 patients to further assess the pharmacokinetics/safety.

Supporting Table S2 Disease characteristics at baseline

Characteristic, *n* (%)	Imaradenant 50 mg QD (*n* = 3)	Imaradenant 75 mg QD (*n* = 7)	Total (*N* = 10)
AJCC disease stage[a]			
I	1 (33)	0	1 (10)
IB	1 (33)	0	1 (10)
IV	0	4 (57)	4 (40)
IVA	0	1 (14)	1 (10)
IVB	0	1 (14)	1 (10)
Missing	1 (33)	1 (14)	2 (20)
AJCC tumor stage			
T1b	2 (67)	0	2 (20)
T2c	0	1 (14)	1 (10)
T3b	0	1 (14)	1 (10)
TX	0	2 (29)	2 (20)
Missing	1 (33)	3 (43)	4 (40)
AJCC node stage			
N0	3 (100)	2 (29)	5 (50)
N1	0	2 (29)	2 (20)
NX	0	1 (14)	1 (10)
Missing	0	2 (29)	2 (20)
AJCC metastasis stage			
M0	3 (100)	2 (29)	5 (50)
M1	0	2 (29)	2 (20)
M1b	0	2 (29)	2 (20)
MX	0	1 (14)	1 (10)
Tumor grade			
Moderately differentiated (G2)	1 (33)	1 (14)	2 (20)
Poorly differentiated (G3)	1 (33)	1 (14)	2 (20)
Unassessable (GX)	1 (33)	2 (29)	3 (30)
High grade	0	2 (29)	2 (20)
Missing	0	1 (14)	1 (10)

Abbreviations: AJCC, American Joint Committee on Cancer; QD, once daily.

[a]AJCC disease stage is based on the initial diagnosis.

Supporting Table S3 Duration of exposure of imaradenant (safety analysis set)

		**Imaradenant 50 mg QD (*n* = 3)**	**Imaradenant 75 mg QD (*n* = 7)**	**Total (*N* = 10)**
Total treatment duration (months)[a]	Mean (SD)	2.21 (1.22)	2.27 (1.46)	2.25 (1.32)
	Median (min–max)	2.10 (1.1–3.5)	2.14 (0.5–4.8)	2.12 (0.5–4.8)
Actual treatment duration (months)[b]	Mean (SD)	2.21 (1.22)	2.17 (1.48)	2.18 (1.34)
	Median (min–max)	2.10 (1.1–3.5)	1.94 (0.3–4.8)	2.00 (0.3–4.8)

Abbreviation: QD, once daily.

[a]Total treatment duration = (last dose date – first dose date + 1) / (365.25 / 12).

[b]Actual treatment duration = total treatment duration, excluding dose interruptions and planned 'no dose' periods for intermittent dosing.

Supporting Table S4 Summary of PK parameters of imaradenant following single and multiple oral administration (PK analysis set)

**PK parameter**	**Summary statistic**	**Imaradenant 50 mg QD (*n* = 3)**	**Imaradenant 75 mg QD (*n* = 7)**
**Single-dose (Cycle 0 Day 1)**	*n*	3	7
C_{max} (ng/mL)	Geometric mean (CV%)	233.9 (29.0)	378.9 (45.6)
t_{max} (h)	Median (min–max)	1.08 (0.95–1.95)	2.00 (0.92–5.52)
$t_{½}$ (h)	Mean (SD)	16.33 (7.067)	18.29 (6.241)
$AUC_{0–24}$ (ng·h/mL)	Geometric mean (CV%)	1825 (28.9)	2926 (30.2)
$AUC_{0–t}$ (ng·h/mL)	Geometric mean (CV%)	2308 (45.0)	3802 (42.2)
AUC (ng·h/mL)	Geometric mean (CV%)	2373 (46.7)	3893 (44.1)
**Multiple-dose (Cycle 1 Day 15)**	*n*	3	6
$C_{max,ss}$ (ng/mL)	Geometric mean (CV%)	314.1 (32.1)	504.6 (66.8)
$t_{max,ss}$ (h)	Median (min–max)	1.97 (1.90–2.07)	1.97 (0.97–3.92)
$AUC_{0–t,ss}$ (ng·h/mL)	Geometric mean (CV%)	2599 (29.6)	4121 (46.1)
$AUC_{0–24,ss}$ (ng·h/mL)	Geometric mean (CV%)	2632 (28.7)	4216 (47.1)
$C_{min,ss}$ (ng/mL)	Geometric mean (CV%)	44.58 (87.7)	64.37 (94.0)
$RacC_{max}$	Geometric mean (CV%)	1.3 (16.1)	1.4 (59.0)
RacAUC	Geometric mean (CV%)	1.4 (5.5)	1.5 (23.1)
TCP	Geometric mean (CV%)	1.1 (17.4)	1.1 (18.4)

Abbreviations: AUC, area under the concentration–time curve; $AUC_{0–24}$, AUC from time 0 to 24 h; $AUC_{0–t}$, AUC up to the last measurable concentration; C_{max}, maximum observed concentration sampled during the dosing interval; CV, coefficient of variation; PK, pharmacokinetic; QD, once daily; RacAUC, accumulation ratio based on AUC; $RacC_{max}$, accumulation ratio based on C_{max}; SS, steady state; TCP, temporal change; $t_{½}$, half-life; t_{max}, time to C_{max}. $RacC_{max}$ was calculated as C_{max} on Cycle 1 Day 15 / C_{max} on Cycle 0 Day 1; RacAUC was calculated as $AUC_{0–24,ss}$ on Cycle 1 Day 15 / $AUC_{0–24}$ on Cycle 0 Day 1; TCP was calculated as $AUC_{0–24,ss}$ on Cycle 1 Day 15 / AUC on Cycle 0 Day 1.

Supporting Table S5 Summary of PK parameters of the active metabolite SSP-005174X in plasma following single and multiple oral administration of imaradenant (PK analysis set)

PK parameter	Summary statistic	Imaradenant 50 mg QD (n = 3)	Imaradenant 75 mg QD (n = 7)
**Single-dose (Cycle 0 Day 1)**	n	3	7
C_{max} (ng/mL)	Geometric mean (CV%)	36.61 (24.0)	37.13 (23.2)
t_{max} (h)	Median (min–max)	1.00 (0.95–1.95)	1.90 (0.92–3.78)
$t_{½}$ (h)	Mean (SD)	18.21 (3.50)	17.83 (5.19)
$AUC_{0–24}$ (ng·h/mL)	Geometric mean (CV%)	263.8 (16.3)	288.0 (24.0)
$AUC_{0–t}$ (ng·h/mL)	Geometric mean (CV%)	338.8 (11.5)	379.4 (30.0)
AUC (ng·h/mL)	Geometric mean (CV%)	348.2 (11.6)	388.2 (30.4)
MP ratio C_{max} (%)	Geometric mean (CV%)	14. 9 (36.2)	9.3 (32.8)
MP ratio AUC (%)	Geometric mean (CV%)	14.0 (39.3)	9.5 (34.7)
**Multiple-dose (Cycle 1 Day 15)**	n	3	6
$C_{max,ss}$ (ng/mL)	Geometric mean (CV%)	44.51 (10.1)	47.30 (73.4)
$t_{max,ss}$ (h)	Median (min–max)	1.97 (1.90–2.07)	1.97 (1.02–7.38)
$AUC_{0–t,ss}$ (ng·h/mL)	Geometric mean (CV%)	325.4 (8.5)	372.5 (45.3)
$AUC_{0–24,ss}$ (ng·h/mL)	Geometric mean (CV%)	331.2 (10.8)	381.2 (46.4)
$C_{min,ss}$ (ng/mL)	Geometric mean (CV%)	5.014 (34.4)	6.051 (95.0)
$RacC_{max}$	Geometric mean (CV%)	1.216 (34.5)	1.333 (51.6)
RacAUC	Geometric mean (CV%)	1.256 (6.3)	1.343 (30.7)
TCP	Geometric mean (CV%)	0.9514 (11.7)	1.000 (16.1)
MP ratio $C_{max,ss}$ (%)	Geometric mean (CV%)	13.5 (27.4)	8.9 (20.0)
MP ratio $AUC_{0–24,ss}$ (%)	Geometric mean (CV%)	12.0 (36.1)	8.6 (14.2)

Abbreviations: AUC, area under the concentration–time curve; $AUC_{0–24}$, AUC from time 0 to 24 h; $AUC_{0–t}$, AUC up to the last measurable concentration; C_{max}, maximum observed concentration sampled during the dosing interval; CV, coefficient of variation; MP, metabolite/parent; PK, pharmacokinetic; QD, once daily; RacAUC, accumulation ratio based on AUC; $RacC_{max}$, accumulation ratio based on C_{max}; SS, steady state; TCP, temporal change; $t_{½}$, half-life; t_{max}, time to C_{max}.

$RacC_{max}$ was calculated as C_{max} on Cycle 1 Day 15 / C_{max} on Cycle 0 Day 1; RacAUC was calculated as $AUC_{0–24,ss}$ on Cycle 1 Day 15 / $AUC_{0–24}$ on Cycle 0 Day 1; TCP was calculated as $AUC_{0–24,ss}$ on Cycle 1 Day 15 / AUC on Cycle 0 Day 1; MP ratio $C_{max,ss}$ (%) was calculated as $C_{max,ss}$ of SSP-005174X / C_{max} of imaradenant on Cycle 1 Day 15 × 100; MP ratio $AUC_{0–24,ss}$ (%) was calculated as $AUC_{0–24,ss}$ of SSP-005174X / $AUC_{0–24,ss}$ of imaradenant on Cycle 1 Day 15 × 100.

Supporting Table S6 Summary of PK parameters of the inactive metabolite SSP-005173X in plasma following single and multiple oral administration of imaradenant (PK analysis set[a])

PK parameter	Summary statistic	Imaradenant 50 mg QD (*n* = 3)	Imaradenant 75 mg QD (*n* = 7)
**Single-dose (Cycle 0 Day 1)**	*n*	3	7
C_{max} (ng/mL)	Geometric mean (CV%)	9.952 (59.3)	7.942 (49.3)
t_{max} (h)	Median (min–max)	2.05 (1.95–5.92)	2.00 (1.85–4.05)
$t_{½}$ (h)	Mean (SD)	14.39 (5.372)	14.55 (6.227)
$AUC_{0–24}$ (ng·h/mL)	Geometric mean (CV%)	115.0 (66.7)	82.39 (47.4)
$AUC_{0–t}$ (ng·h/mL)	Geometric mean (CV%)	158.6 (46.1)	104.5 (53.6)
AUC (ng·h/mL)	Geometric mean (CV%)	171.1 (43.3)	117.4 (50.1)
MP ratio C_{max} (%)	Geometric mean (CV%)	3.9 (87.2)	1.9 (83.7)
MP ratio AUC (%)	Geometric mean (CV%)	6.6 (104.4)	2.8 (49.4)
**Multiple-dose (Cycle 1 Day 15)**	*n*	3	6
$C_{max,ss}$ (ng/mL)	Geometric mean (CV%)	11.94 (39.9)	9.881 (71.5)
$t_{max,ss}$ (h)	Median (min–max)	2.07 (1.90–4.05)	2.09 (1.90–3.92)
$AUC_{0–t,ss}$ (ng·h/mL)	Geometric mean (CV%)	143.2 (73.1)	108.6 (66.3)
$AUC_{0–24,ss}$ (ng·h/mL)	Geometric mean (CV%)	144.0 (74.1)	112.1 (67.7)
$C_{min,ss}$ (ng/mL)	Geometric mean (CV%)	3.104 (47.9)	2.328 (110.2)
$RacC_{max}$	Geometric mean (CV%)	1.200 (22.3)	1.267 (28.4)
RacAUC	Geometric mean (CV%)	1.252 (10.5)	1.329 (34.4)
TCP	Geometric mean (CV%)	0.8417 (25.3)	0.9362 (17.7)
MP ratio $C_{max,ss}$ (%)	Geometric mean (CV%)	3.5 (79.0)	1.8 (59.6)
MP ratio $AUC_{0–24,ss}$ (%)	Geometric mean (CV%)	5.0 (119.4)	2.4 (31.1)

Abbreviations: AUC, area under the concentration–time curve; $AUC_{0–24}$, AUC from time 0 to 24 h; $AUC_{0–t}$, AUC up to the last measurable concentration; C_{max}, maximum observed concentration sampled during the dosing interval; CV, coefficient of variation; MP, metabolite/parent; PK, pharmacokinetic; QD, once daily; RacAUC, accumulation ratio based on AUC; $RacC_{max}$, accumulation ratio based on C_{max}; SS, steady state; TCP, temporal change; $t_{½}$, half-life; t_{max}, time to C_{max}.

$RacC_{max}$ was calculated as C_{max} on Cycle 1 Day 15 / C_{max} on Cycle 0 Day 1; RacAUC was calculated as $AUC_{0–24,ss}$ on Cycle 1 Day 15 / $AUC_{0–24}$ on Cycle 0 Day 1; TCP was calculated as $AUC_{0–24,ss}$ on Cycle 1 Day 15 / AUC on Cycle 0 Day 1; MP ratio $C_{max,ss}$ (%) was calculated as $C_{max,ss}$ of SSP-005173X / C_{max} of imaradenant on Cycle 1 Day 15 × 100; MP ratio $AUC_{0–24,ss}$ (%) was calculated as $AUC_{0–24,ss}$ of SSP-005173X / $AUC_{0–24,ss}$ of imaradenant on Cycle 1 Day 15 × 100.

[a]After database lock, an error was identified in the plasma concentration of SSP-005173X in samples collected at 6 hours post-dose on Cycle 1 Day 15 from one patient. This data point was excluded from calculation of PK parameters.
